# *cpubi4* Is Essential for Development and Virulence in Chestnut Blight Fungus

**DOI:** 10.3389/fmicb.2018.01286

**Published:** 2018-06-15

**Authors:** Qi Chen, Yongbing Li, Jinzi Wang, Ru Li, Baoshan Chen

**Affiliations:** ^1^State Key Laboratory of Conservation and Utilization of Subtropical Agro-Bioresources, Nanning, China; ^2^College of Life Science and Technology, Guangxi University, Nanning, China

**Keywords:** polyubiquitin, virulence, ubiquitination, hypovirus, chestnut blight fungus

## Abstract

Ubiquitination plays key roles in eukaryotic growth, stress adaptation, and metabolic regulation. In our previous work, ubiquitin was found to be secreted in the hypovirus-infected strain of *Cryphonectria parasitica*, a phytopathogenic filamentous fungus responsible for the chestnut blight. Here we report the functional and molecular characterization of a polyubiquitin gene, *cpubi4*, in *C. parasitica*. The expression of *cpubi4* was upregulated by the infection of a hypovirus. Deletion of *cpubi4* resulted in abnormal morphology, reduced sporulation, attenuation of virulence, and significant reduction in ubiquitination. A total of 378 sites in 236 proteins were identified to be significantly decreased in ubiquitination in the absence of *cpubi4*. Quantitative proteome analysis revealed that 285 in 4,776 identified proteins changed in abundance (1.5-fold, *P* < 0.05) in the *cpubi4* null mutant, as compared with the wild-type strain.

## Introduction

Ubiquitin is a highly conserved 76-amino acid protein, produced through *de novo* synthesis by ubiquitin coding genes and recycled from the ubiquitinated proteins by deubiquitinating enzymes (Kimura and Tanaka, 2010). Ubiquitin mediates post-translational modifications of proteins and plays critical roles in a variety of cellular processes, including transcriptional regulation, signal transduction, cell cycle, and cellular differentiation (Finley et al., 1987; Hershko and Ciechanover, 1998). There are four ubiquitin-encoding genes in mammal, *UBA52* and *RPS27A* encode a single copy ubiquitin fused to ribosomal proteins L40 and S27a, respectively, and the other two genes encode polyubiquitin precursors with different number of ubiquitin repeats (Kimura and Tanaka, 2010). In yeast, there are three ubiquitin-related genes (*UBI1, UBI2*, and *UBI3*) each encoding a single ubiquitin that is fused to unrelated amino acid sequences (Ozkaynak et al., 1987) and the fourth gene *UBI4* encodes a polyubiquitin precursor containing five ubiquitin repeats in a head-to-tail manner (Ozkaynak et al., 1984). In *Candida albicans*, inactivation of polyubiquitin gene *UBI4* affected fungal growth, stress resistance ability, and virulence (Leach et al., 2011). In rice blast pathogenic fungi *Magnaporthe oryzae*, deletion of polyubiquitin-encoding gene MGG_01282, resulted in a reduction of growth and sporulation, abnormal conidia, reduced germination and appressorium formation, and lost the ability to cause disease (Oh et al., 2012).

*Cryphonectria parasitica*, a filamentous fungus, is the causative agent of chestnut blight, well known for its destructive effect on the chestnut forestry in North America. Hypoviruses are a group of plus-stranded RNA virus originally found in the chestnut blight fungus, known for its ability to attenuate the virulence of its host (termed hypovirulence) (Nuss, 2005). In addition to hypovirulence, hypovirus-infected *C. parasitica* strains exhibit a phenotype different from the virus-free wild-type strain, i.e., reduction in pigmentation, inhibition of sporulation, and female infertility. By virtue of transmissible hypovirulence, hypovirus-carrying strains have been used as biocontrol agents for chestnut blight disease (Anagnostakis, 1982). Due to the availability of genetic manipulation system for both the virus and the host fungus, hypovirus-*C. parasitica* interaction has evolved into a model to dissect the mechanism of virulence regulation in pathogenic fungi (Nuss, 2005; Eusebio-Cope et al., 2015). In our previous secretome analysis, ubiquitin was found to be up-regulated in the hypovirus-infected strain EP155/CHV1-EP713, indicating that ubiquitin expression may be manipulated by the hypovirus (Wang et al., 2016). To better understand the roles of ubiquitin in *C. parasitica*, we disrupted a polyubiquitin-encoding gene, *cpubi4*, and analyzed the phenotypic traits of the mutants. The *cpubi4* mutants were impaired in development with diminished orange pigment, slower growth, reduced sporulation, and attenuated virulence. Ubiquitomics analysis revealed that *cpubi4* was responsible for ubiquitination of a wide range of proteins, some of which are of critical importance in biological and metabolic processes.

## Materials and methods

### Fungal strains and growth conditions

*C. parasitica* EP155 (ATCC38755) is an orange pigment-producing, virulent, and highly sporulated wild-type strain. EP155/CHV1-EP713 is isogenic to EP155 but harboring hypovirus CHV1-EP713 by transfection with a synthetic infectious viral transcript (Chen et al., 1994). EP155/CHV1-EP713 is white, hypovirulent, and produces hardly any conidial spore on both PDA (Difco, Detroit, USA) or chestnut stems. DK80 was derived from EP155 but with its nonhomologous end joining DNA repair component encoding gene *ku80* deleted. DK80 is highly efficient in gene homologous replacement, indistinguishable from the parental wild-type strain EP155 in colony morphology, conidiation, virulence, and ability to support hypovirus replication (Lan et al., 2008; Choi et al., 2012). All strains, including the *cpubi4* deletion strains, were maintained on PDA at 25°C with a 12-h light at 1,500 lx and 12-h dark cycle (Hillman et al., 1990).

### Gene manipulation

Preparation of protoplast and transformation of *C. parasitica* were carried out essentially as described previously (Churchill et al., 1990). The antibiotics hygromycin B (40 μg/ml) or G418 (25 μg/ml) was added to the medium for selection of transformants.

#### Deletion of the *cpubi4* gene in *C. parasitica*

Disruption of *C. parasitica* gene was performed by homologous recombination. Primers and templates used to generate relevant fragments were listed in Supplementary Table 1. The 780-bp left flanking fragment, the 2146-bp *hph* cassette and the 761-bp right flanking fragment were fused to form a 3.7-kb cassette by double-joint PCR with 1:3:1 molar ratio. The PCR reaction cycles consisted of 94°C for 2 min, followed by 15 cycles of 94°C for 30 s, 58°C for 2 min, and 72°C for 4 min, with a final extension of 5 min at 72°C. A nested PCR was performed following the double-joint PCR using the primer pair *cpubi4*-LF/*cpubi4*-RR. After verification by agarose gel electrophoresis and gel extraction, 500 ng of *hph* cassette was used to transform DK80 protoplasts as described previously (Lan et al., 2008). Putative *cpubi4* disruptants were identified by PCR, purified to nuclear homogeneity by single-spore isolation and further verified by Southern blot analysis. The confirmed transformants were designated as Δ*cpubi4* strains. Gene cloning, PCR, and Southern analysis were performed as described (Sambrook and Russell, 2001).

#### Complementation of *cpubi4* mutant

To construct the complementary strains for Δ*cpubi4*, a 4-kb genomic fragment containing the complete *cpubi4* transcript region (983 bp), promoter region (1.6 kb), and terminator region (1.2 kb) from EP155 DNA was amplified and cloned into *SacII* and *HpaI* sites of vector pCPXG418 to generate plasmid pCPXG418/*cpubi4*. The *cpubi4* complementary strains were obtained by transforming Δ*cpubi4* protoplast with plasmid pCPXG418/*cpubi4*. Selected transformants were subjected to single-spore purification with antibiotic G418 as the selection marker. PCR was performed to confirm the complementation of *cpubi4* gene.

### DNA and RNA analysis

The fungal strains were grown in EP complete medium (Puhalla and Anagnostakis, 1971) for 3 days at 25°C. DNA or RNA was prepared from the cultures as described (Shi et al., 2014). Total cDNA was generated with oligo (dT) and random primer, using a RevertAid RT Reverse Transcription Kit (Thermo Scientific). PCRs were performed in a Light Cycler 480 (Roche Applied Science). Relative accumulation level of a gene transcript was examined by quantitative real-time RT-PCR using the 18S rRNA values to normalize for variations in template concentrations (Livak and Schmittgen, 2001).

### Protein analysis

#### Protein preparation and quantification

Fungal mycelia were ground into powder in liquid nitrogen and re-suspended in 1 ml of pre-chilled acetone (−20°C) containing 10% TCA and 0.07% β-mercaptoethanol. After incubation at −20°C for 2 h, the mixture was centrifuged at 13,523 g for 30 min at 4°C. The pellet was washed with ice-cooled acetone, air-dried, and then re-suspended in lysis buffer (7.5 M urea, 2.5 M thiourea, 12.5% glycerol, 50 mM Tris, and 2% protease inhibitor; Wang et al., 2014). After treated with ultrasonic at 200 W, the protein sample was centrifuged at 13,523 g for 30 min at 4°C to remove the debris. Three replicates for each strain were performed. The protein concentration was determined using Bradford Protein Assay (Bio-Rad, Hercules, CA, USA). The quality of the protein preparations was checked with SDS-PAGE using a Mini-PROTEAN system (BioRad, Hercules, USA) by loading 30 μg of protein per lane. After separation by electrophoresis, the gels were stained with Coomassie solution.

#### Western blotting

Simple Western system (Simon™, ProteinSimple, San Jose, CA) was used to analyze protein ubiquitination (Chen et al., 2013). The concentration of protein lysates was adjusted at 20 μg/μl with RIPA buffer (Beyotime Institute of Biotechnology, Shanghai, China) and 4 μl of the adjusted lysate (containing 80 μg of protein) was mixed with a master mix to a final concentration of 1 × sample buffer, 1 × fluorescent molecular weight marker, and 40 mM DTT. The ubiquitination of total proteins was identified using ubiquitin-specific antibody (catalog #sc-8017, Santa Cruz, USA) as primary antibody at 1:50 dilution. Compass software (ProteinSimple) was used to analyze the captured digital image.

### Protein ubiquitination analysis

#### Ubiquitinated peptide affinity enrichment

For trypsin digestion, the protein solution was reduced with 10 mM DTT for 2.5 h at 37°C and alkylated with 50 mM IAA for 30 min at room temperature in darkness. An amount of 5 mg of protein sample was digested with trypsin, and label-free immune-affinity enrichment was performed using di-Gly-Lys antibody (Cell Signaling Technology), which is specifically recognizes the di-Gly-Lys remnant of ubiquitinated peptides (Udeshi et al., 2013). The protein samples were diluted by adding an appropriate volume of water to a urea concentration of <2 M. Trypsin was added at a 1:50 (trypsin: protein) mass ratio for digestion overnight. Digested peptides were desalted using a C18 SPE column (Waters), and freeze drying. Digested peptides were dissolved with precooled IAP buffer (50 mM MOPS/NaOH pH 7.2, 10 mM Na_2_HPO_4_, 50 mM NaCl), and then incubated with prewashed Anti-K-ε-GG antibody beads (PTM Biolabs) at 4°C overnight with gentle shaking. The beads were washed 3 times with 1 ml precooled IAP buffer and twice times with 1 ml precooled ddH_2_O. The bound peptides were eluted from the di-Gly-Lys antibody beads with 0.1% trifluoroacetic acid at room temperature twice for 10 min. The two elute fractions were combined, and desalted with C18 STAGE Tips (Thermo Fisher), then vacuum-dried, and thawed with 0.1% formic acid (Rappsilber et al., 2007). Three biological replicates were performed.

#### TMT labeling

The labeling was done following manufacturer's protocol. One hundred micro grams of digested peptides were labeled with TMT kit (Thermo Fisher Scientific, USA). A 6-plex TMT-126,−127,−128,−129,−130, and−131 were used to label protein samples of DK80 and Δ*cpubi4*, with three independent biological replicates respectively. Labeled peptides were mixed at equal ratios and subjected to high-pH reverse-phase HPLC using an Agilent 300 Extend C18 column (5 μm particles, 4.6 mm i.d., 250 mm length) for fractionation. Fractions were collected with a gradient of 2–60% acetonitrile, and dried by vacuum centrifugation, then thawed with 0.1% formic acid for further LC-MS/MS analysis.

#### LC-MS/MS analysis

The peptides were analyzed using LC-MS/MS according to previously described protocols (Li et al., 2015). Briefly, peptides passed through a reverse-phase analytical column were loaded into the reverse-phase precolumn (EASY column SC001; Thermo Scientific, USA) that was connected to a reverse-phase column (EASY column SC200; Thermo Scientific, USA), separated with a linear gradient of solvent B (0.1% formic acid in 80% acetonitrile) at a constant flow rate of 300 nL/min on an EASY-nLC 1000 UPLC system. The separated peptides were analyzed with the Q Exactive hybrid quadrupole-Orbitrap mass spectrometer (Thermo Fisher Scientific, USA). Intact peptides were detected on the Orbitrap at a resolution of 70,000. Peptides of the top 20 most abundant precursor ions from the survey scan (300–1,800 m/z) were selected for HCD fragmentation. The ion fragments were detected on the Orbitrap at a resolution of 17,500. Determination of the target value was based on predictive automatic gain control.

### Database search

For characterization of ubiquitinated peptides, the MS data were processed using MaxQuant software (version 1.3.0.5) and searched against the *C. parasitica* v2 database. The enzymatic cleavage rule was trypsin allowing a maximum of four missed cleavage sites. First, the search range was set at 5 ppm for precursor ions, and the main search range was set at 6 ppm and 0.02 D for fragment ions. Carbamidomethylation on cysteines was defined as a fixed modification. Oxidation on methionine, acetylation on protein N-terminal and ubiquitination on Lysine were defined as variable modifications for database searching. The cutoff for the global false discovery rate for peptides was set at 0.01. The intensity of the monoisotopic parent ion (precursor) was used to quantify each peptide (peptide intensity from MaxQuant software). Quantification was based on spectral characteristics (retention time, m/z ratio, and peak intensity) determined by comparing the direct mass spectrometric signal intensities for given peptides.

For characterization of proteomic peptides, the RAW data were processed using Mascot software (version 2.2) and Proteome Discoverer (version 1.4) and searched against the *C. parasitica* v2 database. The enzymatic cleavage rule was trypsin allowing a maximum of two missed cleavage sites. The mass tolerance was set to 20 ppm for the peptides and 0.1 Da for the fragment. Carbamidomethyl on Cys, TMT-6 plex on N-terminal, and TMT-6 plex on Lys were specified as fixed modifications, and oxidation on Met and TMT-6 plex on Tyr was specified as a variable modification. A decoy database search for determining false discovery rate (FDR) was set for a maximum of 1%. The protein ratios were calculated as the median of unique peptides of the protein.

### Biological assays

#### Temperature stress assays

Temperature stress assays were done by keeping the 3-days-old fungal cultures on PDA at 4°C for 60 days or at 37°C for 7 days. After the treatment, the strains were re-inoculated onto PDA at 25°C to examine the survival rate. Three replicates were executed.

#### Virulence assays

Virulence assays were performed on stems of Chinese chestnut with three replicates per fungal strain as previously described (Yao et al., 2013). Fungal strains were cultured on PDA plates for 5 days to allow the hyphal colony to grow to about 3–5 cm in diameter. For inoculation, the bark in the stem was removed with a 5-mm cork borer (about 3 mm deep), and a 5-mm plug with fungal mycelium from the PDA plate was inserted into the shallow hole. Inoculated stems were kept at room temperature in a plastic bag to maintain moisture. Canker statistical analysis was performed with the Proc GLM procedure SAS (version 8.0), and the type I error rate was set at 0.05.

### Bioinformatics

The protein sequence of yeast UBI4 (NP_013061) was used to BLAST *C. parasitica* genome^1^ for searching the ubiquitin coding gene. The protein conserved domain analyses were conducted using the NCBI database^2^. Statics were based on three independent experiments, and Student's test was used to compare the difference of means between two strains (Wang et al., 2016).

The Kyoto Encyclopedia of Genes and Genomes (KEGG) online tool KAAS was used to annotate protein pathways (Kanehisa and Goto, 2000). The annotated proteins were mapped on the KEGG pathway using the KEGG Mapper. KOBAS software was used to test the statistical enrichment of the proteins in KEGG pathways (Wu et al., 2006).

## Results

### Identification of ubiquitin-coding genes in *C. parasitica*

**Figure 1:**
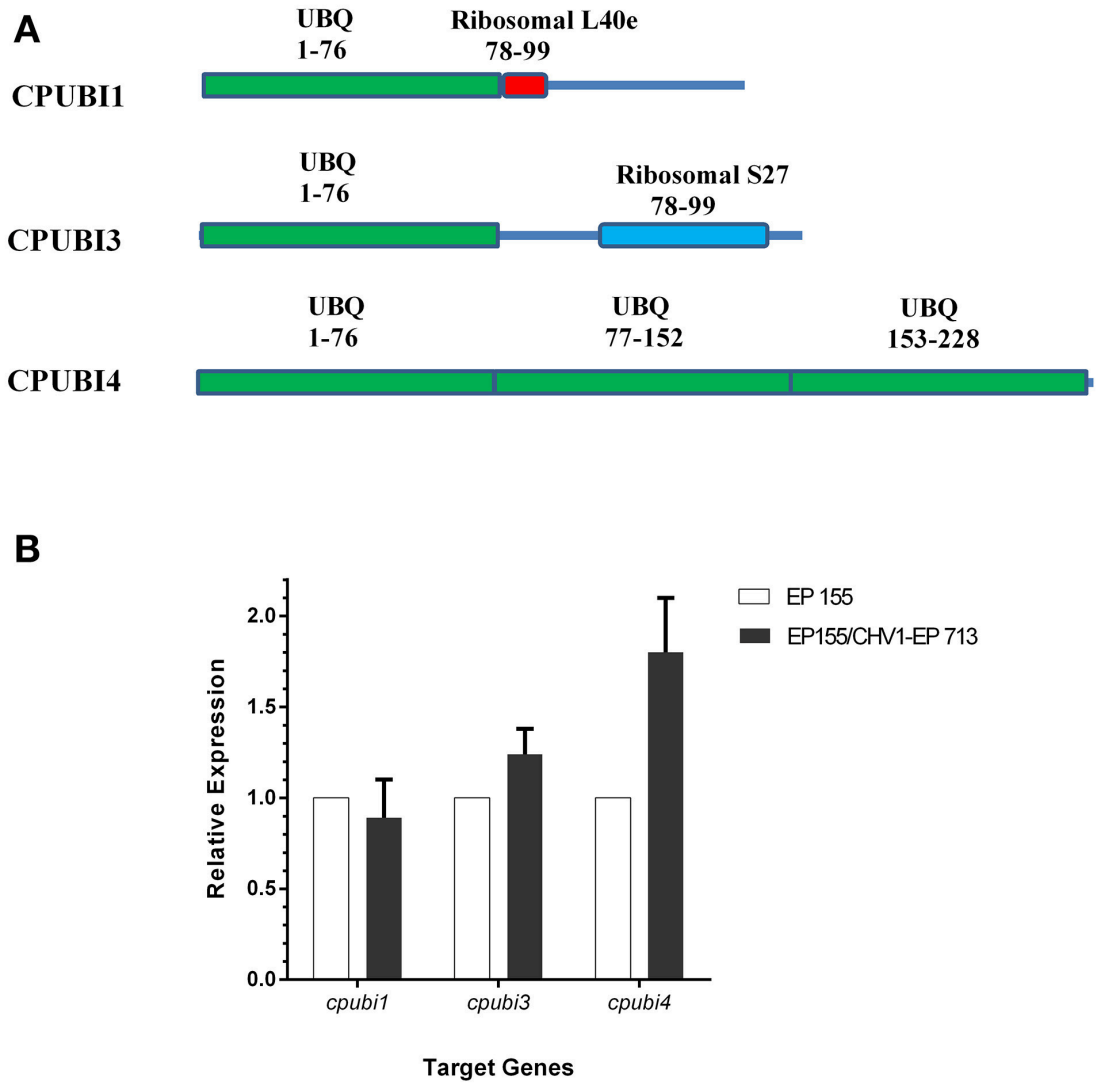
Schematic representation of ubiquitin coding genes in *C. parasitica* and quantification of transcript accumulation levels of ubiquitin coding genes in response to infection by hypovirus CHV1-EP713. **(A)** The UBQ domains were identified by searching NCBI's Conserved Domains database and are indicated by green boxes; Ribosomal L40e domain in CPUBI1 is indicated by the red box; Ribosomal S27 domain in CPUBI3 is indicated by the blue box. Solid lines represent full-length proteins. The ranges of amino acids included in the match are indicated next to and below the domain name. **(B)** Strains were cultured on PDA for 7 days, and total RNA was extracted. The expression of *cpubi1, cpubi3*, and *cpubi4* transcripts was measured by quantitative real-time RT-PCR (2^−ΔΔ*CT*^ method). The relative transcript level was calibrated using 18S rRNA as reference, and the transcript levels of EP155 were set to a value of one (Lin et al., 2007). Three replicates were performed.

Ubiquitin is highly conserved in amino acids sequence (Ozkaynak et al., 1987). A secreted protein spot was identified as ubiquitin with increased accumulation in CHV1-EP713 infected strain in our previous work (Wang et al., 2016). To identify ubiquitin gene candidates, we used the 76 aa protein sequence of yeast ubiquitin protein (NP_013061) to blast against EP155 genome. Three ubiquitin motif-containing genes were identified in *C. parasitica*, designated as *cpubi1* (scaffold_3:3613558-3614685), *cpubi3* (scaffold_6:1195287-1196660), and *cpubi4* (scaffold_8:305148-306130). Analysis of conserved domain with CD-search Tool at NCBI^2^ revealed that CPUBI1 contains a UBQ superfamily motif in position 1–76 and a ribosomal L40 family motif in position 78–100; CPUBI3 contains a UBQ superfamily motif in position 1–76 and a ribosomal S27 family motif in position 103–147; CPUBI4 contains three UBQ superfamily in positions 1–76, 77–152, and 153–228, respectively (Figure 1A). The *cpubi1* and *cpubi3* encode hybrid proteins in which ubiquitin is fused to other amino acid sequences. The third ubiquitin gene, *cpubi4* encodes a polyubiquitin with three consecutive head-to-tail ubiquitin monomers. To investigate whether the transcription of ubiquitin genes are affected by the infection of CHV1-EP713, the wild-type strains EP155 and the hypovirus infected strain EP155/CHV1-EP713 were examined for the transcript accumulation of the three ubiquitin genes by quantitative real-time RT-PCR. In 7-days-old cultures on PDA, expression of *cpubi1* and *cpubi3* were not significantly altered upon viral infection. By contrast, *cpubi4* expression was up-regulated by about 80% in hypovirus infected strain (Figure 1B).

### Knockout of *cpubi4* affects *C. parasitica* phenotypes

**Figure 2:**
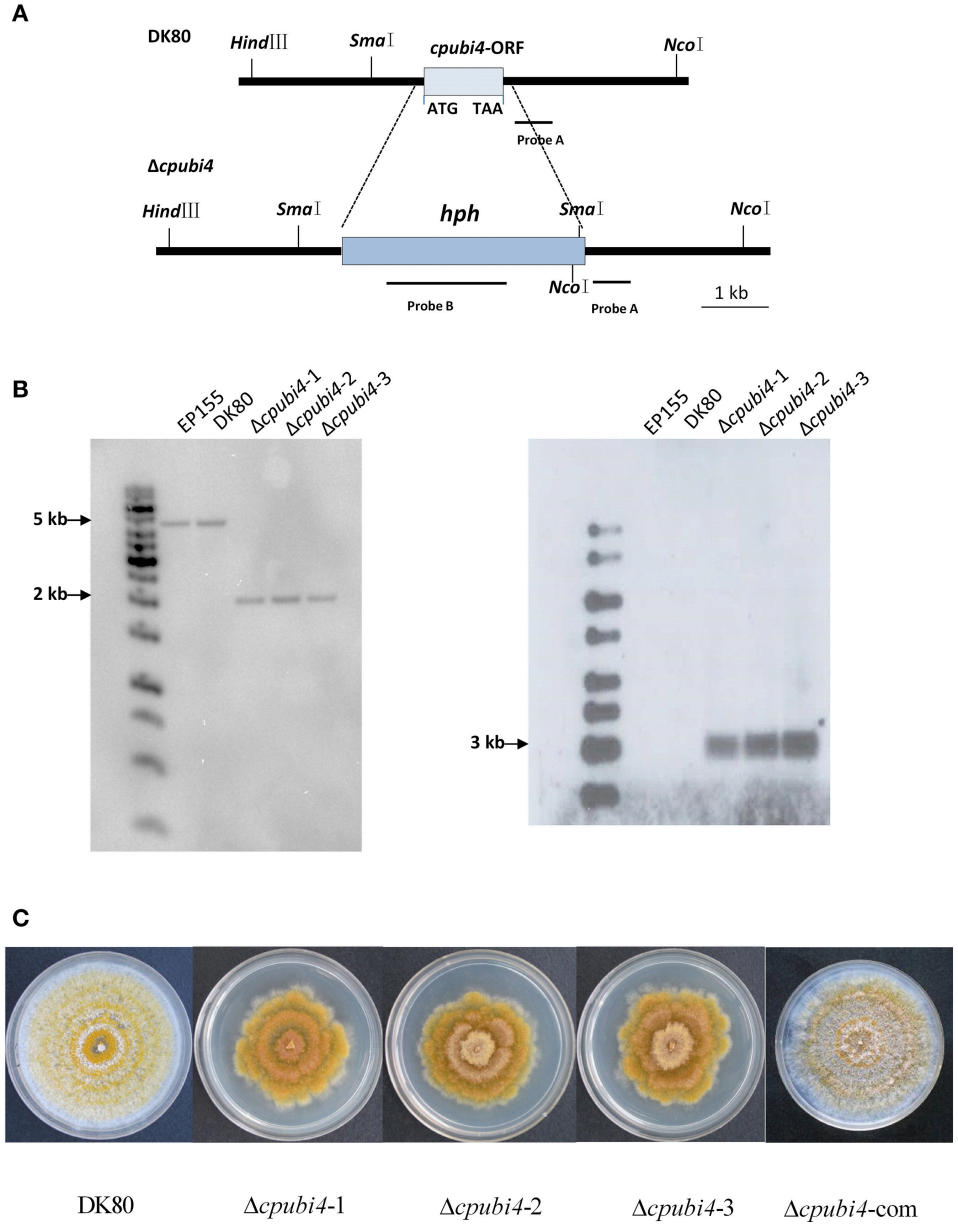
Southern blot analysis and phenotypes of *C. parasitica cpubi4* gene disruption mutants. **(A)** The genomic DNA containing the *cpubi4* coding region composed of one exon is indicated by the white box. **(A)** *cpubi4* gene replacement construct was generated by insertion of the 3.6 kb hygromycin (*hph*) resistant cassette using homologous recombination strategy (see section Materials and Methods). Probes fragment on the right arm and hph were used in the Southern blot analysis to distinguish the wild-type strain and *cpubi4* null mutants. **(B)** Southern analysis of *cpubi4* disruptant strains. DNA samples were digested with *Hind*III/*Nco*I (left), or independently with *Sma*I (right), and blotted using the probe A, and probe B, respectively. Fragments sizes are indicated in the figure margins. **(C)** Colony morphologies of DK80, Δ*cpubi4*, and the *cpubi4* complementary strain Δ*cpubi4*-com on PDA plates.

**Figure 3:**
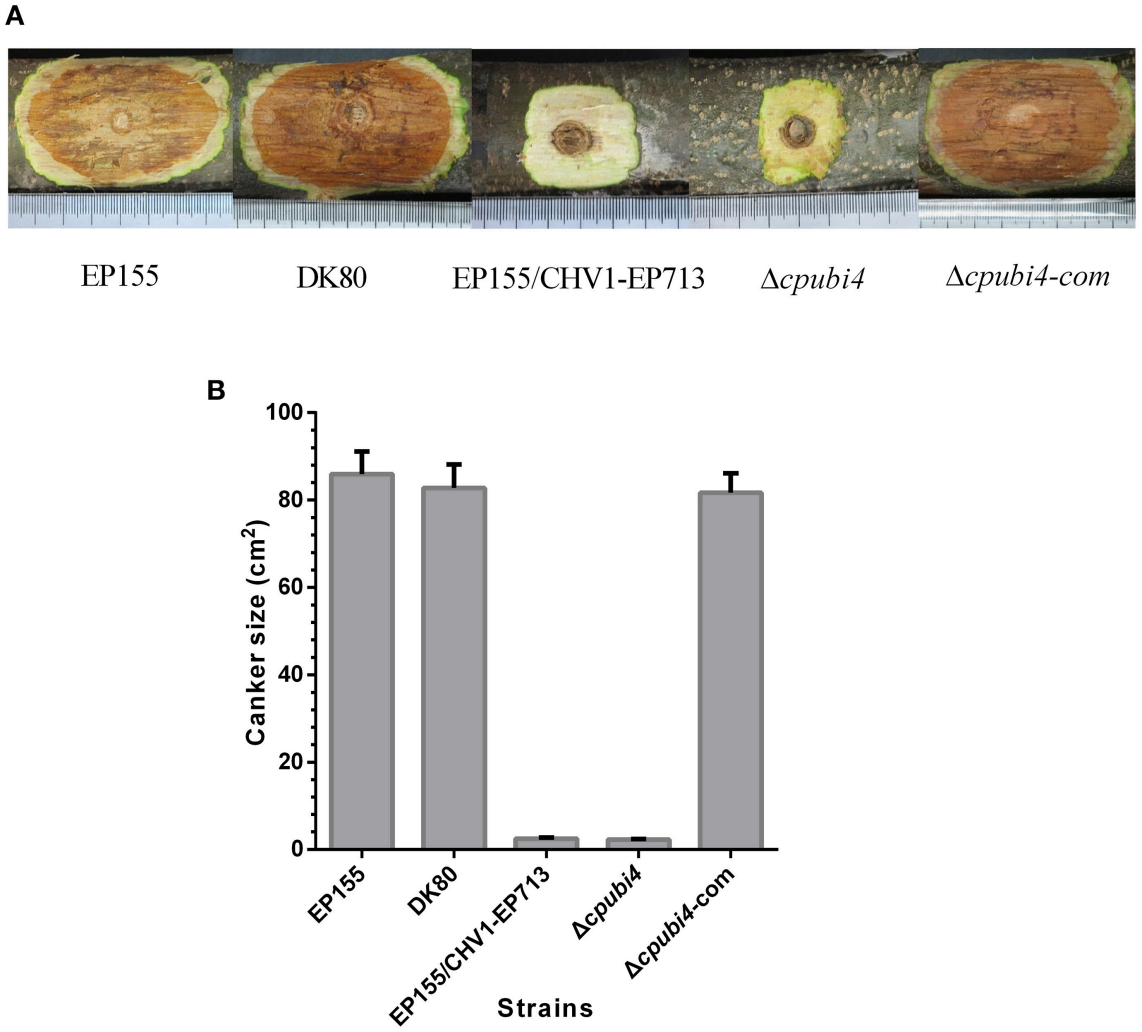
Virulence assay on chestnut stems. **(A)** Cankers caused by the tested strains. The wild-type strain EP155, starting strain DK80, hypovirus-infected EP155/CHV1-EP713, *cpubi4* disrupts strain Δcpubi4, and *cpubi4*-complemented strain Δ*cpubi4*-com were inoculated onto Chinese chestnut (*Castanea mollissima* Blume) stems. The inoculated stems were kept at 24°C. The resulting cankers were photographed and measured at day 21 post-inoculation. **(B)** Statistical analysis of virulence of the *cpubi4* null mutants. The assays were with three duplicates for each strain.

To investigate the function of *cpubi4*, we designed a gene replacement cassette with hygromycin B resistance as a selection marker to disrupt *cpubi4* via homologous recombination (Figure 2A). Three randomly selected single-spore derived transformants, Δ*cpubi4-1*, Δ*cpubi4-2*, and Δ*cpubi4-3*, were further confirmed by Southern blot analysis (Figure 2B). The growth of Δ*cpubi4* mutants was slower, and the aerial hyphae were significantly less than the wild-type strain and the parental strain DK80. The conidiation level was also drastically reduced. The altered phenotype of the mutants could be fully restored by re-introducing a wild-type copy of *cpubi4* (Figure 2C). More importantly, Δ*cpubi4* lost the ability to incite a canker on the chestnut stem, similar to the hypovirulent strain EP155/CHV1-EP713. Again, the virulence could be restored to the wild-type level after a wild-type copy of *cpubi4* was reintroduced into the Δ*cpubi4* mutant (Figure 3).

### *cpubi4* is essential for temperature stress adaption but does not affect hypovirus dsRNA accumulation in *C. parasitica*

**Figure 4:**
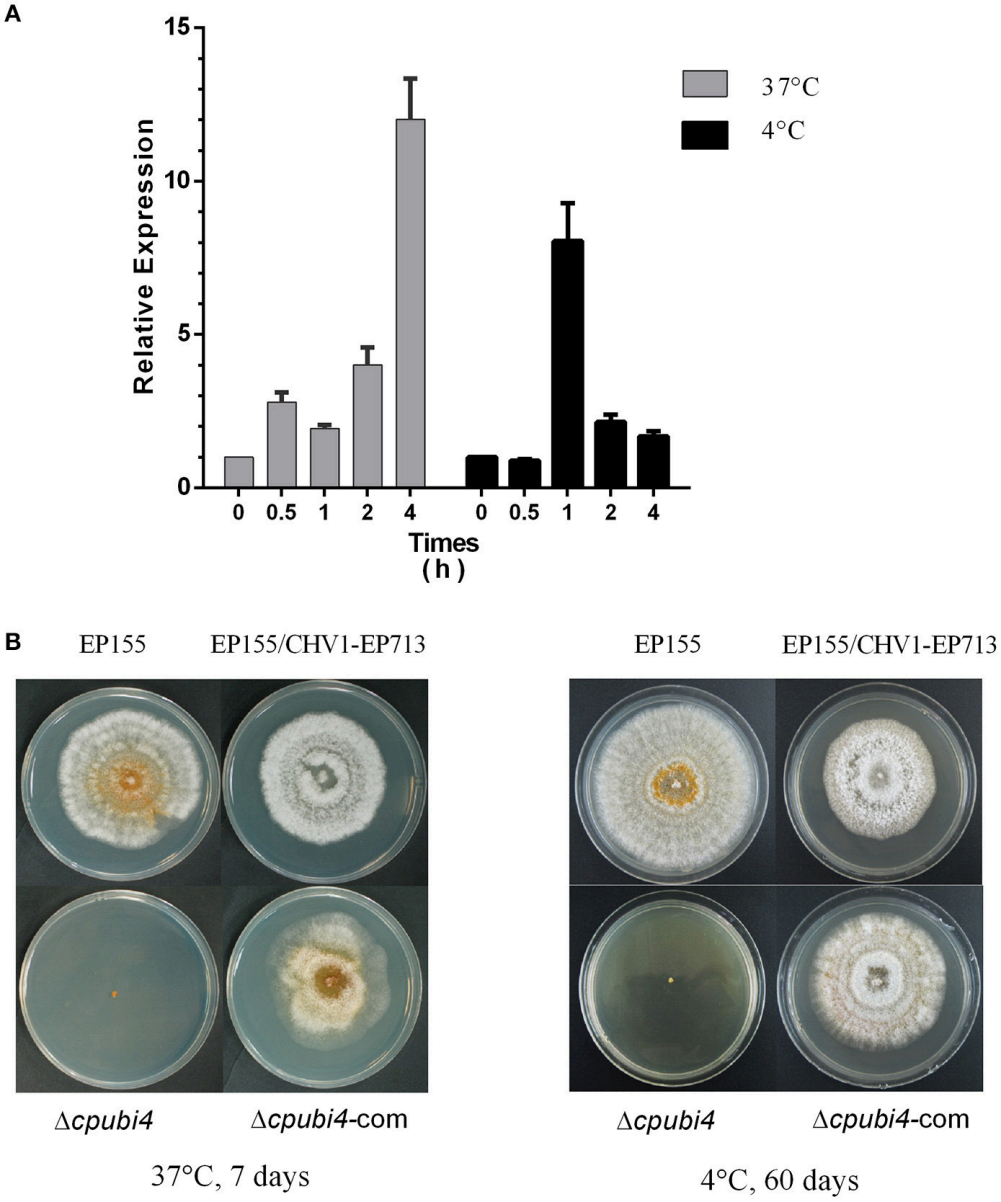
Response of *C. parasitica* strains to temperature stress. **(A)** Quantification of *cpubi4* transcript. EP155 was first cultured on PDA plates at 25°C for 3 days, and then transferred to 37°C or 4°C incubators for certain periods of time. RNA quantification was done as above (see legend to Figure 1). *cpubi4* transcript level at time zero was set at a value of 1.0. **(B)** Viability and morphology of the strains. After being kept at 37°C for 1 week or 4°C for 2 months, the strains were inoculated onto PDA plate at 25°C. Photographs were taken at day 7 post-inoculation.

In *S. cerevisiae, UBI4* expression was increased in response to elevated temperatures, and inactivation of *UBI4* resulted in sensitivity to high-temperature stress (Finley et al., 1987). We reasoned that the *cpubi4* gene might contribute to the temperature stress adaption in *C. parasitica*. To test this hypothesis, we used real-time RT-PCR to analyze the transcript accumulation of *cpubi4* gene in the wild-type strain EP155 after heat and cold treatments. EP155 was first cultured on PDA plates at 25°C for 3 days, then kept at 37 and 4°C, respectively. After treatments, the strains were harvested, and total RNA was isolated for real-time RT-PCR. It was found that the expression of *cpubi4* was upregulated for more than 2-fold after 37°C treatment for 0.5 h, and increased to more than 10-fold after for 4 h. Similarly, the expression of *cpubi4* was also increased by 5-fold after cold treatment at 4°C for 1 h (Figure 4A). To further confirm the roles of *cpubi4* in temperature adaption, we tested the viability of wild-type strain EP155, virus-harboring EP155/CHV1-EP713, Δ*cpubi4*, and Δ*cpubi4*-com under temperature stress conditions. The Δ*cpubi4* failed to grow after treatment at 37°C for 1 week and at 4°C for 2 months, respectively. In contrast, EP155, EP155/CHV1-EP713, and Δ*cpubi4*-com were viable under the same conditions (Figure 4B). Collectively, our data indicate that polyubiquitin gene *cpubi4* is required for temperature adaptation in *C. parasitica*.

To verify if the *cpubi4* gene would influence the accumulation of the hypovirus, we introduced CHV1-EP713 into Δ*cpubi4* via anastomosis by paring hypovirus-infected strain EP155/CHV1-EP713 with Δ*cpubi4*. Infection by the hypovirus resulted in the loss of orange pigment in Δ*cpubi4*, but the mutant seemed to support viral replication almost equally well as the wild-type strain (Supplementary Figure 1).

### *cpubi4* contributes significantly to the global ubiquitination in *C. parasitica*

**Figure 5:**
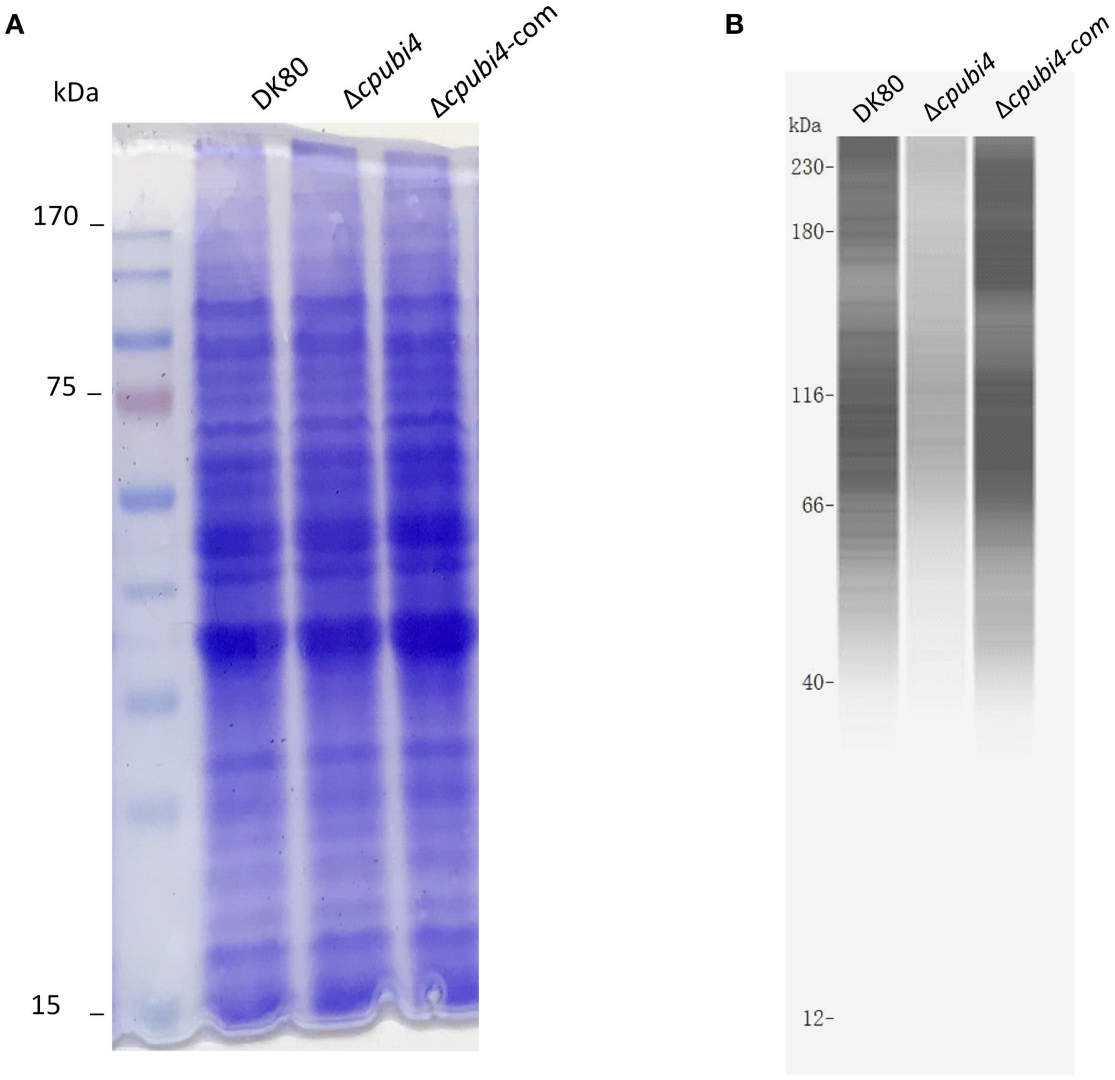
Evaluation of the ubiquitome of wild-type DK80 and Δ*cpubi4* strains. **(A)** SDS-PAGE. Loading amount was 30 μg of total protein extracted from 3-days old culture in EP. The gel was stained with Coomassie solution. **(B)** Immuno blot analysis on a Simple Western system with ubiquitin-specific antibody. An amount of 80 μg of total protein was loaded for each lane. Signals were stronger with the high molecular weight proteins, likely due to multi ubiquitination sites in the protein. Δ*cpubi4*-com represents the wild-type *cpubi4*-complemented Δ*cpubi4* strain.

With the same amount of mycelia (0.8 g), Δ*cpubi4* yielded 33.9% more total protein than its parental strain Dk80 (6.87 vs. 5.13 mg, paired Student's test *p* = 0.009). To evaluate the global effects of polyubiquitin on proteins ubiquitination, total proteins from the wild-type and *cpubi4*-deleted strains were analyzed by Western blot with ubiquitin antibody. With the same amount of protein loaded, the ubiquitination proteins in the Δ*cpubi4* were strikingly less than its parental DK80 (Figure 5). We also compared the global ubiquitination of the wild-type strain EP155 and the hypovirus-infected strain EP155/CHV1-EP713 and found that hypovirus infection led to slightly enhanced ubiquitination (Supplementary Figure 2).

**Figure 6:**
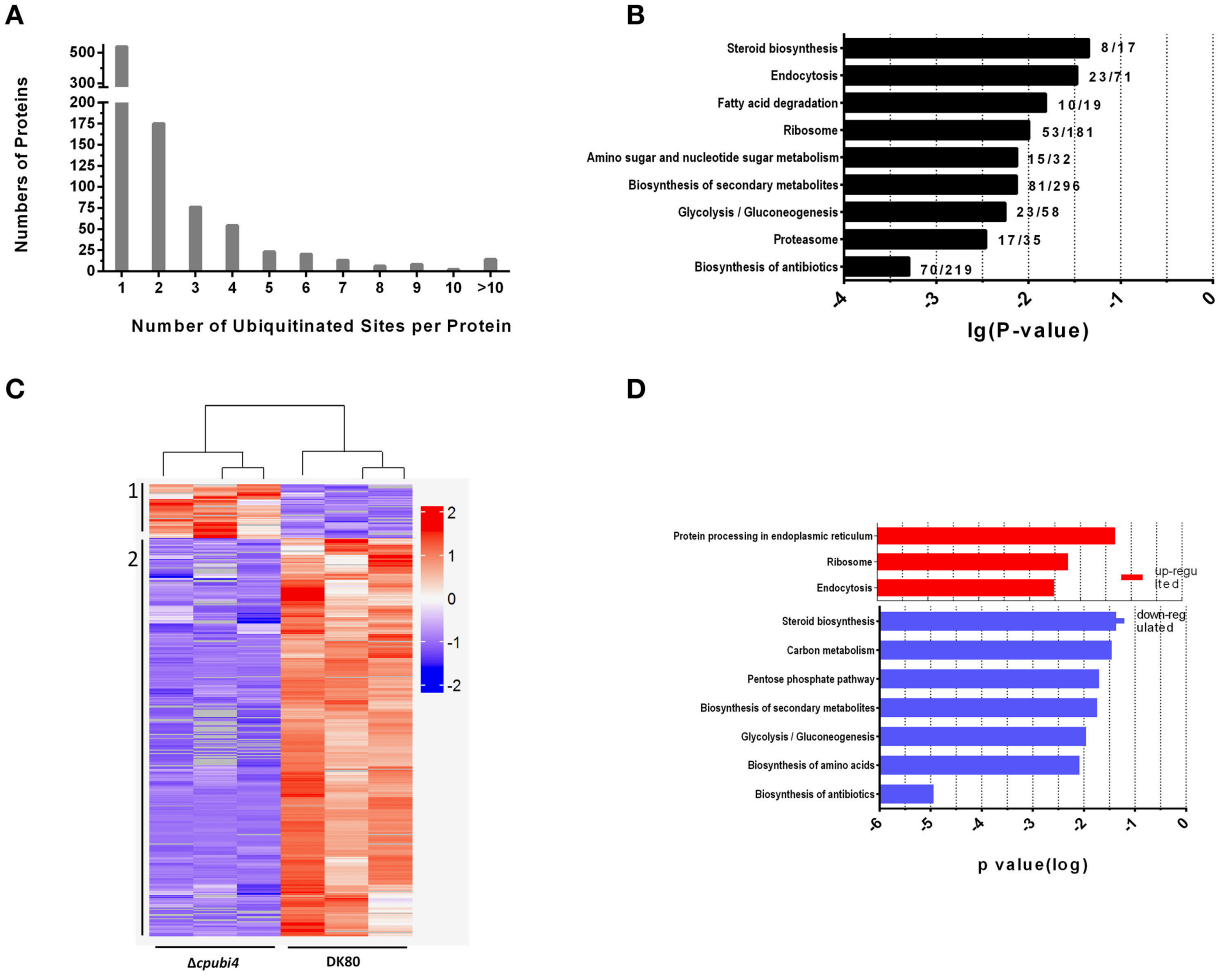
Characteristics of ubiquitylome of *C. parasitica*. **(A)** Distribution of ubiquitination sites identified per protein. **(B)** KEGG pathway enrichment of proteins from DK80. Significance was ranked according to the *p*-value. **(C)** Heat-map of the hierarchical clusters of the 429 affected ubiquitinated peptides (fold change > 2, *p*-value < 0.05) in the mutant stains Δ*cpubi4*. Peptides showing similar expression patterns have been clustered. For details about peptides ubiquitination, see the Supplementary Table 2. **(D)** KEGG pathway enrichment of proteins with up-regulated (red) and down-regulated (blue) ubiquitination sites. Significance was ranked according to the *p*-value.

To quantify the ubiquitinated proteins in *C. parasitica*, a label-free quantitative strategy involving antibody-based affinity enrichment and high-resolution LC-MS/MS were employed for both DK80 and Δ*cpubi4* in parallel. In three independent experiments, a total of 2,684 di-Gly-Lys ubiquitination sites that mapped to 1195 unique proteins were identified in DK80 and Δ*cpubi4* (Supplementary Table 2). Between 1 and 24 putative ubiquitination sites, with the majority of one site (57.9%) and two sites (18.8%), were found in proteins from DK80 (Figure 6A). The most ubiquitinated proteins were hypothetical proteins (id: 330586, 331295) with 24 and 22 lysine ubiquitination sites, and the 70 kDa heat shock protein (HSP70) with 17 lysine ubiquitination sites. Majority of the ubiquitinated proteins were enriched in pathways of key cellular processes, including metabolism of protein, fatty acid, and sugar, as well as second metabolites (Figure 6B).

To identify protein substrates of the polyubiquitin, we compared the abundance of di-Gly-Lys-containing peptides between Δ*cpubi4* and DK80 (Supplementary Table 3). A total of 428 proteins with ubiquitination level change >2-fold (*p* < 0.05) were identified, among which 50 peptides in 45 proteins were increased, and 378 peptides in 236 proteins decreased in ubiquitination (Figure 6C). Among these proteins, some are essential metabolic enzymes, including enolase, peptidyl-prolyl cis-trans isomerase (CYP1); mitogen-activated protein kinases, ribosome, G-protein, ubiquitin-related protein; and stress responding such as heat shock proteins (Table 1). KEGG enrichment showed that proteins with increased ubiquitination function mainly in protein synthesis and processing and endocytosis, while proteins with decreased ubiquitination function in biosynthesis of amino acid, steroid, antibiotics, and secondary metabolites; and in carbon metabolism, glycolysis/gluconeogenesis, and pentose phosphate pathway (Figure 6D).

**Table 1:** Examples of proteins with significant changes in ubiquitination.

**Proteins ID**	**Proteins description**	**Up-regulated ubiquitination sites^a^**	**Down-regulated ubiquitination sites^b^**	**Positions within proteins^c^**
**HEAT SHOCK PROTEIN**				
227872	Heat shock protein sti1 homolog	–	1	152
331015	Heat shock protein SSB1	–	2	326,967
253316	Heat shock protein hsp88	1	–	755
245307	Heat shock protein 90 homolog	–	4	86,235,384,567
260193	Heat shock 70 kDa protein 12A	–	1	732
346270	Heat shock 70 kDa protein	3	5	323,326,450,499,511,523,556,560
**MAPK PATHWAY**				
262898	1,3-beta-glucan synthase component FKS1	–	1	951
231325	Mitogen-activated protein kinase	–	1	279
277770	Peptidyl-prolyl cis-trans isomerase, mitochondrial	–	2	129,211
**UBIQUITIN-MEDIATED PROTEOLYSIS**				
108221	E3 ubiquitin-protein ligase RSP5	–	3	87,88,780
258058	Ubiquitin-conjugating enzyme E2 N	–	2	81,91
328806	Ubiquitin-activating enzyme E1 1	–	2	568,569
245142	Ubiquitin-conjugating enzyme E2-34 kDa	–	1	15
334719	NEDD8-conjugating enzyme UBC12	–	1	98
331066	Ubiquitin-conjugating enzyme E2-20 kDa	1	–	16
328046	Ubiquitin-conjugating enzyme E2 14	–	1	94
**UBIQUITIN**				
103787	Ubiquitin-40S ribosomal protein S27a	–	4	6,29,48,63
331210	Ubiquitin-like protein pmt3/smt3	–	1	120
263247	Ubiquitin-like protein 1	1	–	45
**PROTEASOME**				
334850	Probable proteasome subunit beta type-4	–	1	221
333003	Probable proteasome subunit alpha type-7	1	–	64
263113	26S proteasome regulatory subunit RPN10	–	1	133
107783	26S proteasome non-ATPase regulatory subunit 1	–	1	1059
220665	26S protease regulatory subunit 8 homolog	–	1	20
220595	Probable 26S protease subunit rpt4	–	2	26,198
**G-PROTEIN**				
325103	Guanine nucleotide-binding protein subunit gamma	–	1	84
103579	Guanine nucleotide-binding protein subunit beta-like protein	–	1	280
**RIBOSOME**				
223537	60S ribosomal protein L9-B	–	1	84
105502	60S ribosomal protein L4-A	–	1	174
333723	60S ribosomal protein L36	–	1	50
103417	60S ribosomal protein L30	–	1	86
271891	60S ribosomal protein L17	1	1	52,96
281725	60S ribosomal protein L13	–	1	53
283348	60S ribosomal protein L12	–	1	48
280826	60S ribosomal protein L11	–	1	35
222795	40S ribosomal protein S3	–	4	78,168,200,205
103717	40S ribosomal protein S20	–	5	8,15,33,45,50
103050	40S ribosomal protein S2	–	1	39
242837	40S ribosomal protein S18	1	–	53
273821	40S ribosomal protein S15	1	–	85
329021	40S ribosomal protein S17	–	2	544,634
224177	40S ribosomal protein S13	–	3	27,39,43
271207	40S ribosomal protein S12	1	1	56,94
101666	40S ribosomal protein S10-B	–	2	93,137
103787	Ubiquitin-40S ribosomal protein S27a	–	4	6,29,48,63
**GLYCOLYSIS/GLUCONEOGENESIS**				
104720	Fructose-bisphosphate aldolase	–	1	115
252290	Pyruvate decarboxylase	–	2	40,523
258754	Pyruvate kinase	–	8	43,99,148,246,268,426,483,516
106275	Alcohol dehydrogenase 1	–	1	31
328301	Phosphoglycerate kinase	–	7	15,17,83,91,150,156,295
249839	ATP-dependent 6-phosphofructokinase	–	3	352,638,690
101684	Glyceraldehyde-3-phosphate dehydrogenase	–	4	185,193,214,332
221435	Alcohol dehydrogenase	–	1	211
233295	Triosephosphate isomerase	–	3	144,175,216
103155	Enolase	–	7	60,64,79,96,120,194,347

*A portion of proteins with significant changes in ubiquitination due to lack of cpubi4*.

a *Number of up-regulated ubiquitination sites from the protein*.

b *Number of down-regulated ubiquitination sites from the protein*.

c *The position of ubiquitination site in the protein*.

### Deletion of *cpubi4* alters protein expression patterns

**Figure 7:**
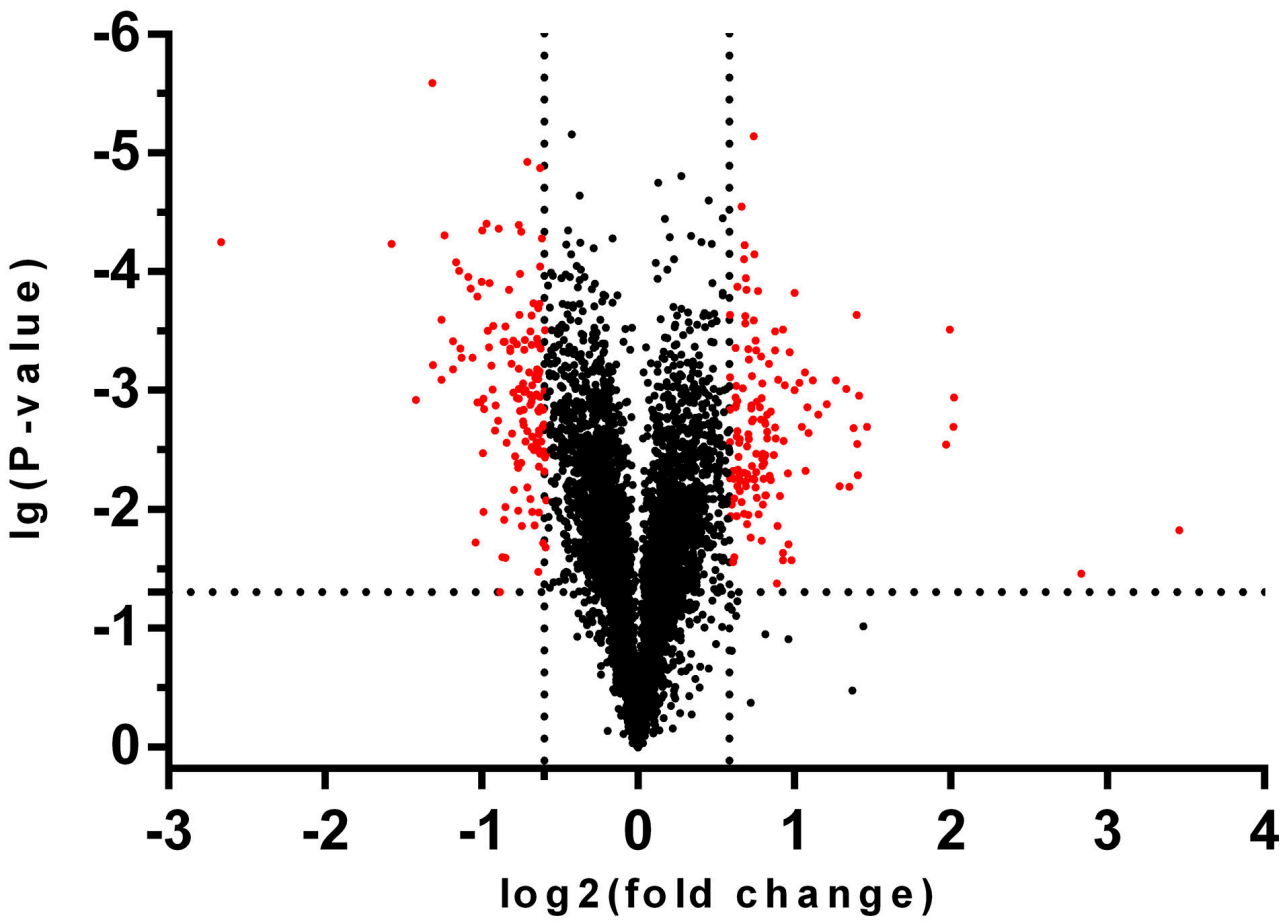
Volcano plot of the proteomes of DK80 and Δ*cpubi4*. Black dots represent the total proteins identified from both DK80 and Δ*cpubi4*, red dots represent the differentially expressed proteins in Δ*cpubi4* with fold-change greater than 1.5 and *p* < 0.05. A total of 142 proteins exhibited reduced ubiquitination upon deletion of cpubi4, indicating that they are the substrates of CPUBI4.

To see if deletion of *cpubi4* would change the abundance of specific proteins, we quantified the proteomes of Δ*cpubi4* and DK80 using the same amount of total protein with three biological replicates. Among 4,767 proteins quantified, the vast majority of proteins remained basically unchanged (< 1.5-fold; Supplementary Table 4). Volcano plotting of proteomes of both strains identified a subset of proteins with fold-change ≥1.5 and *p*-value < 0.05 (Figure 7). As seen in Table 2, the up-regulated proteins contain many ubiquitin-conjugating enzymes and ubiquitin-protein ligases. Other up-regulated proteins include glutathione transporter, methyltransferases, serine/threonine- and mitogen-activated protein kinases, mitochondrial activity proteins (cytochrome P450/NADPH-P450), autophagy-related protein, and apoptosis-inducing factor 2. Among the down-regulated proteins, are many ribosomal proteins, belonging to 30S, 40S, and 60S. Besides, mitochondrion-related proteins, e.g., mitochondrial ribosomal protein, mitochondrial import inner membrane translocase subunits, and NADH-ubiquinone oxidoreductase subunit were also down-regulated (Table 2, Supplementary Table 4 for detail information). Accumulation of ubiquitin-conjugating enzymes was a sign of speeding up in ubiquitin-recycling process and reduction of essential mitochondrial proteins would jeopardize the normal function of a mitochondrion.

**Table 2:** Examples of proteins up-regulated and down-regulated by deletion of *cpubi4*^a^.

**Protein ID^b^**	**Proteins description**	**Average ratio^c^**	***t* test *p*-value^d^**
**UP-REGULATED PROTEINS**			
**Ubiquitin-Conjugating Enzyme**			
255535	Ubiquitin-conjugating enzyme E2Q-like protein CG4502	1.737895085	0.0042126
331066	Ubiquitin-conjugating enzyme E2-20 kDa	1.7595107	0.00755426
232237	Ubiquitin-conjugating enzyme E2 J1	1.611945028	0.000112666
243167	Ubiquitin-conjugating enzyme E2 8	1.736645576	0.00335787
104034	Ubiquitin-conjugating enzyme E2 6	2.305704848	0.00129594
252753	Ubiquitin-conjugating enzyme E2 2	2.168058757	0.000809489
328046	Ubiquitin-conjugating enzyme E2 14	1.648350773	0.0171935
**Ubiquitin-Protein Ligase**			
215338	E3 ubiquitin-protein ligase ubr1	1.56805089	0.00254144
274970	E3 ubiquitin-protein ligase itt1	1.567666682	0.00697192
341211	E3 ubiquitin-protein ligase dbl4	1.75861228	0.00343743
266035	Probable HECT-type ubiquitin ligase-interacting protein creD	1.609215843	0.00026883
274707	STIP1 homology and U box-containing protein 1	1.57155688	0.00569711
335876	PHD and RING finger domain-containing protein C126.07c	2.100016891	0.00467357
255228	F-box protein YLR352W	1.68577	0.000456609
**Transport Activity**			
223105	Vesicle-associated membrane protein 7B	1.667315817	0.000254997
252500	Transmembrane protein 184 homolog C30D11.06c	1.50120028	0.00144419
220118	UDP-galactose transporter homolog 1	1.702008993	0.000144171
325360	Store-operated calcium entry-associated regulatory factor	1.651085956	0.0013294
109037	Probable ran guanine nucleotide release factor	1.670196023	7.16E-06
**Transferase Activity**			
345836	Transaldolase	1.732496465	0.00370331
245522	Sterol 24-C-methyltransferase	1.758109157	0.00752994
330996	Probable S-adenosylmethionine-dependent methyltransferase CRG1	2.044572993	0.000850226
109461	Lovastatin diketide synthase LovF	1.501137033	0.00269576
351527	GDP-Man:Man(3)GlcNAc(2)-PP-Dol alpha-1,2-mannosyltransferase	1.501428385	0.00539262
219562	Histone-lysine N-methyltransferase, H3 lysine-79 specific	1.730819125	0.0011541
**Kinase**			
261928	Mitogen-activated protein kinase 20	1.915753416	0.000893821
66486	Serine/threonine-protein kinase ATG1	1.561655632	0.00359346
**Others**			
229417	Bifunctional cytochrome P450/NADPH–P450 reductase	1.746372739	0.00361005
219948	Apoptosis-inducing factor 2	1.839557188	0.00251934
277081	Autophagy-related protein 8	1.684164422	0.00647872
**DOWN-REGULATED PROTEINS**			
**Ribosomal-Related Protein**			
332673	60S ribosomal protein L35	0.662046014	0.0036187
333723	60S ribosomal protein L36	0.65764289	0.0191438
102781	60S ribosomal protein L14	0.638642957	0.0006562
103140	60S acidic ribosomal protein P0	0.632333414	0.0134515
244514	40S ribosomal protein S29	0.618094491	0.0006993
224760	60S acidic ribosomal protein P1	0.6063268	0.0003723
100780	60S ribosomal protein L7	0.585235713	0.0011423
234544	40S ribosomal protein S19	0.577554815	0.0067663
275342	Ubiquitin-60S ribosomal protein L40	0.502721352	0.003331
358141	30S ribosomal protein 2, chloroplastic	0.552951761	0.0121389
103787	Ubiquitin-40S ribosomal protein S27a	0.571426883	0.0005912
**Mitochondrial Protein**			
235111	Mitochondrial import inner membrane translocase subunit TIM9	0.655945111	0.0020106
225024	Mitochondrial 37S ribosomal protein S27	0.643542957	0.0014632
328740	Altered inheritance of mitochondria protein 41, mitochondrial	0.592556243	0.0001034
327534	Delta-1-pyrroline-5-carboxylate dehydrogenase, mitochondrial	0.591234659	0.0002282
357354	Respiratory supercomplex factor 1, mitochondrial	0.588303102	0.0101486
220172	Probable proline dehydrogenase, mitochondrial	0.626096845	0.0024221
221383	Probable 3-hydroxyisobutyrate dehydrogenase, mitochondrial	0.58799909	0.0044118
224403	ATP synthase subunit f, mitochondrial	0.586069963	0.0040819
107757	ATP synthase subunit 4, mitochondrial	0.53900636	4.32E-05
245314	Mitochondrial import inner membrane translocase subunit TIM13	0.532395384	0.0013224
329791	Cytochrome c oxidase polypeptide 5, mitochondrial	0.644503601	0.0043218
334855	Cytochrome c oxidase subunit 6, mitochondrial	0.643009812	0.0001996
238062	2-methylcitrate dehydratase, mitochondrial	0.644348666	0.0023069
220455	2-oxoisovalerate dehydrogenase subunit beta, mitochondrial	0.644098628	0.0105008
**NADH Related Protein**			
261324	NADH-cytochrome b5 reductase 2	0.650784426	0.0026476
224496	NADH dehydrogenase	0.638523013	0.000362
322879	NADH-ubiquinone oxidoreductase 10.5 kDa subunit	0.628958467	0.0001824

a *See Supplementary Table 4 for the complete list*.

b *Accession number from Cryp80honectria parasitica database v2.0*.

c *Parental stain DK80 was set as reference. The average ratio (Δcpubi4/DK80) were from three independent experimental data*.

d *Scored using Student's t-test, n = 3, It could be used to see if they are significantly different from each other by student's test*.

Cross-checking of the differentially accumulated proteins with the ubiquitome identifies 79 proteins that show weak correlation between protein abundance and number of ubiquitination site per protein: among the up-regulated proteins in Δ*cpubi4*, 85.7% of them contain 1 or 2 ubiquitination sites, no protein contains more than 5 ubiquitination sites; while among the down-regulated proteins, 65.9% contain 1 or 2 ubiquitination sites, but 25% contain 5 or more ubiquitination sites (Table 3). However, since proteins identified from the ubiquitome are far fewer than those from the proteomes (about 1/4), the exact correlation between protein degradation profile and the ubiquitination sites in a protein remains to be verified in the future.

**Table 3:** Number of ubiquitination site in differentially accumulated proteins^a^.

**Protein ID**	**Description**	**Number of ubiquitination site^c^**
**UP-REGULATED PROTEINS^b^**		
246699	–	4
340305	–	3
331066	Ubiquitin-conjugating enzyme E2-20 kDa	3
245522	Sterol 24-C-methyltransferase	3
277081	Autophagy-related protein 8	3
280355	30 kDa heat shock protein	2
349821	Uncharacterized ATP-dependent helicase C23E6.02	2
341211	E3 ubiquitin-protein ligase dbl4	2
329877	Protein SPT23	2
282187	ATPase family AAA domain-containing protein 3B	2
328046	Ubiquitin-conjugating enzyme E2 14	2
108749	L-ornithine N(5)-monooxygenase	2
266035	Probable HECT-type ubiquitin ligase-interacting protein creD	2
269206	Transcription elongation factor 1	2
325438	Carboxypeptidase Y homolog A	2
326539	–	1
344892	–	1
329908	–	1
103600	–	1
104034	Ubiquitin-conjugating enzyme E2 6	1
252753	Ubiquitin-conjugating enzyme E2 2	1
75354	SWIRM domain-containing protein YOR338W	1
261928	Mitogen-activated protein kinase 20	1
271551	Aldehyde dehydrogenase	1
267009	Cell division control protein 4	1
255535	Ubiquitin-conjugating enzyme E2Q-like protein CG4502	1
243167	Ubiquitin-conjugating enzyme E2 8	1
107003	–	1
219053	Protein ssh4	1
223105	Vesicle-associated membrane protein 7B	1
333459	–	1
332318	–	1
248650	NAD-dependent protein deacetylase SRT1	1
252500	Transmembrane protein 184 homolog C30D11.06c	1
109461	Lovastatin diketide synthase LovF	1
**DOWN-REGULATED PROTEINS**		
330586	–^d^	33
67838	–	16
337355	14-3-3 protein homolog	10
226763	Probable 5-methyltetrahydropteroyltriglutamate–homocysteine methyltransferase	9
103787	Ubiquitin-40S ribosomal protein S27a	8
104921	–	7
101014	–	7
267236	Indoleamine 2,3-dioxygenase 1	7
324752	–	6
253168	Glycogen phosphorylase	5
275342	Ubiquitin-60S ribosomal protein L40	5
332673	60S ribosomal protein L35	4
332324	–	4
105888	–	3
102597	Uncharacterized protein C9E9.04	3
103866	–	2
103140	60S acidic ribosomal protein P0	2
105467	Farnesyl pyrophosphate synthase	2
263597	Putative HC-toxin efflux carrier TOXA	2
332325	–	2
103157	Putative daunorubicin C-13 ketoreductase DnrU	2
328973	–	2
245581	Lipid phosphate phosphatase 1	2
332047	Uncharacterized protein MT3735	2
333723	60S ribosomal protein L36	1
325606	–	1
102781	60S ribosomal protein L14	1
245734	Levodione reductase	1
263730	–	1
335178	–	1
102161	–	1
244514	40S ribosomal protein S29	1
100780	60S ribosomal protein L7	1
234544	40S ribosomal protein S19	1
250085	Elongation factor 1-gamma-A	1
333867	Homoserine O-acetyltransferase	1
251895	Lysine acetyltransferase	1
265885	Versicolorin B synthase	1
253380	–	1
105308	Uncharacterized protein C32A11.02c	1
326707	Tetracycline resistance protein from transposon Tn4351/Tn4400	1
255109	2,3-dimethylmalate lyase	1
255822	Uncharacterized oxidoreductase C162.03	1
343596	Uncharacterized protein MT1298	1

a *The proteins were differentially regulated by deletion of cpubi4*.

b *For details of up-regulated and down-regulated proteins, see Supplementary Table 4*.

c *For details information of ubiquitination sites, see Supplementary Table 2*.

d *-means the protein is an unknown protein*.

## Discussion

### Hypovirus infection specifically up-regulates *cpubi4* expression

There are three ubiquitin-encoding genes in *C. parasitica*, but only *cpubi4* responds to the infection of a hypovirus at transcription level (Figure 1). This observation provides a line of evidence that hypovirus perturbs the host protein ubiquitination through regulation of transcription of a specific ubiquitin-encoding gene. A similar observation was reported in *S. cerevisiae* in which *UBI4* was the only one among four ubiquitin-encoding genes to respond to stresses (Fraser et al., 1991), and in pea plant in which a heat-inducible polyubiquitin gene was induced by the infection of pea seed-borne mosaic virus (Aranda et al., 1996).

It has been reported that viruses hijack the host ubiquitin system to enhance viral replication, through modification of host ubiquitination to degrade the cellular proteins, or recruiting ubiquitin to modify viral proteins (Randow and Lehner, 2009). To test if CPUBI4 is required for the hypovirus replication, we introduced CHV1-EP713 into a *cpubi4*-null mutant. However, deletion of *cpubi4* did not seem to have a significant impact on the accumulation of the hypoviral dsRNA (Supplementary Figure 1), suggesting that the increase of *cpubi4* expression is not for the replication of hypovirus, but a result of responding to the hypovirus infection. Since there are two other ubiquitin genes in *C. parasitica* genome, the involvement of ubiquitin in the replication of hypovirus remains to be clarified. On the other side, increased accumulation of *cpubi4* transcript did not seem to lead to the significant increase in ubiquitination of the host proteins (Supplementary Figure 2), indicating that hypovirus infection does not alter ubiquitination pattern at a global basis. Since the interplay between viruses and ubiquitin system are complex (Calistri et al., 2014), how hypovirus bypasses or exploits the ubiquitin system still needs further investigation.

### CPUBI4 is a major contributor to *C. parasitica* ubiquitination

It was reported that deletion of polyubiquitin gene *Ubc* in mouse embryonic fibroblasts resulted in a reduction of total ubiquitin by 40% (Ryu et al., 2007). Western blotting with ubiquitin and polyubiquitin-specific antibody revealed that the total ubiquitinated proteins were dramatically reduced in Δ*cpubi4* (Figure 5), and comparative ubiquitome analysis further showed that *cpubi4* is the major player for ubiquitination in *C. parasitica* (Supplementary Table 2). The fact that total amount of protein in mycelia is higher in Δ*cpubi4* mutant than in the wild-type strain also suggests that lack of sufficient amount of ubiquitin may result in an elevated level of cellular protein. We assume that reduction in ubiquitination level in Δ*cpubi4* might slow down the protein degradation process, resulting in accumulation of proteins in the cells.

### Mechanism of CPUBI4 regulation of development, stress adaptation, and virulence in *C. parasitica*

As a protein fate controller, ubiquitin modulates gene function by control of gene product quantity. Ubiquitin homeostasis, or the maintenance of free ubiquitin above the certain threshold level, is important for cellular function and survival under normal or stress conditions (Park and Ryu, 2014). In fungi, the polyubiquitin-encoding gene is not indispensable but is required for development and adaption to environmental changes. For example, loss of UBI4 in *S. cerevisiae* tenders the fungus to be more sensitive to starvation, amino acid analogs, and high temperatures (Finley et al., 1987; Tanaka et al., 1988; Fraser et al., 1991). Deletion of *UBI4* in *C. albicans* resulted in altered morphology, defects in cell cycle, and loss of virulence (Leach et al., 2011). In *M. oryzae*, the polyubiquitin deletion mutant exhibited defects in morphology and virulence (Oh et al., 2012). Likewise, *cpubi4* gene was shown to be required for mycelial growth, sporulation, virulence, and temperature stress adaption (Figures 2–4).

Previous study had shown that metabolic enzymes were targets of ubiquitination in *C. albicans* (Leach et al., 2011) and in the current study, a similar conclusion was reached (Supplementary Table 3). It has been demonstrated that CYP1, MAPK2, MAPK1 are components of or related to the heterotrimeric G-protein signaling pathway and required for growth, pigmentation, conidiation, and virulence in *C. parasitica* (Park et al., 2004; Choi et al., 2005; Chen et al., 2011). Although slower growth in Δ*cpubi4* may lead to reduction in virulence and sporulation, alteration in ubiquitination level of key proteins may also contribute to virulence attenuation and lower level of sporulation in Δ*cpubi4*, as it has been noticed that sporulation and virulence are regulated by different mechanisms in *C. parasitica* (Yao et al., 2013).

Heat shock proteins (Hsps) that function as molecular chaperones are crucial in adaptation to harsh conditions such as temperature stress for an organism (Richter et al., 2010). Several Hsps including Hsp70, Hsp90, STI1, and SSB1 were found to be at a significantly lower ubiquitination level in Δ*cpubi4* (Supplementary Table 3). It is speculated that change in ubiquitination of these heat shock proteins could account for the temperature stress sensitivity in Δ*cpubi4* (Figure 4). In this regard, polyubiquitin deletion has been shown to affect growth, morphogenesis, and virulence in *C. albicans* and *Aspergillus fumigatus* (Lorenz and Fink, 2001; Hwang et al., 2003; Bhabhra and Askew, 2005).

Another dimension that CPUBI4 may have an impact on is to regulate cellular protein level. As found in comparative proteome analysis, a small fraction of proteins (288 in 4767) changed in abundance due to lack of CPUBI4 (Supplementary Table 4). These include some important proteins that function in protein synthesis and degradation, enzymatic activity, mitochondrial activity, and energy generation (Table 3). Imbalanced accumulation of proteins in the cells may disturb the orchestra of cellular proteins.

In conclusion, we demonstrated that *cpubi4* is the major ubiquitin gene that contributes significantly to the global ubiquitination in the chestnut blight fungus and this gene plays crucial roles in the growth, conidial development, temperature adaptation, and virulence in *C. parasitica*. Knowledge gained through this study provides new insights into the mechanism of *cpubi4* in regulation of multiple traits of *C. parasitica*.

## Author contributions

QC, JW, RL, and BC conceived and designed the experiments. QC and YL performed the experiments. QC analyzed the data and wrote the manuscript draft. BC supervised the project and wrote the manuscript.

### Conflict of interest statement

The authors declare that the research was conducted in the absence of any commercial or financial relationships that could be construed as a potential conflict of interest.

## Footnotes

^1^https://genome.jgi.doe.gov/pages/blast-query.jsf?db=Crypa2

^2^https://www.ncbi.nlm.nih.gov/Structure/cdd/wrpsb.cgi
