# Supplementary material for: cpubi4 Is Essential for Development and Virulence in Chestnut Blight Fungus
Source: Front Microbiol. 2018 Jun 15;9:1286. doi: 10.3389/fmicb.2018.01286 (PMC6013588; doi:10.3389/fmicb.2018.01286)
Supplement: Supplementary file 5 [file Data_Sheet_1.DOCX]

***cpubi4* is essential for development and virulence in chestnut blight fungus**

Qi Chen^1,2,^ Yongbing Li^2^, Jinzi Wang^1,2^, Ru Li^1, 2^, Baoshan Chen^1, 2^ *

* Correspondence: Prof. Baoshan Chen, chenyaoj@gxu.edu.cn

## 1. Supplementary Figures


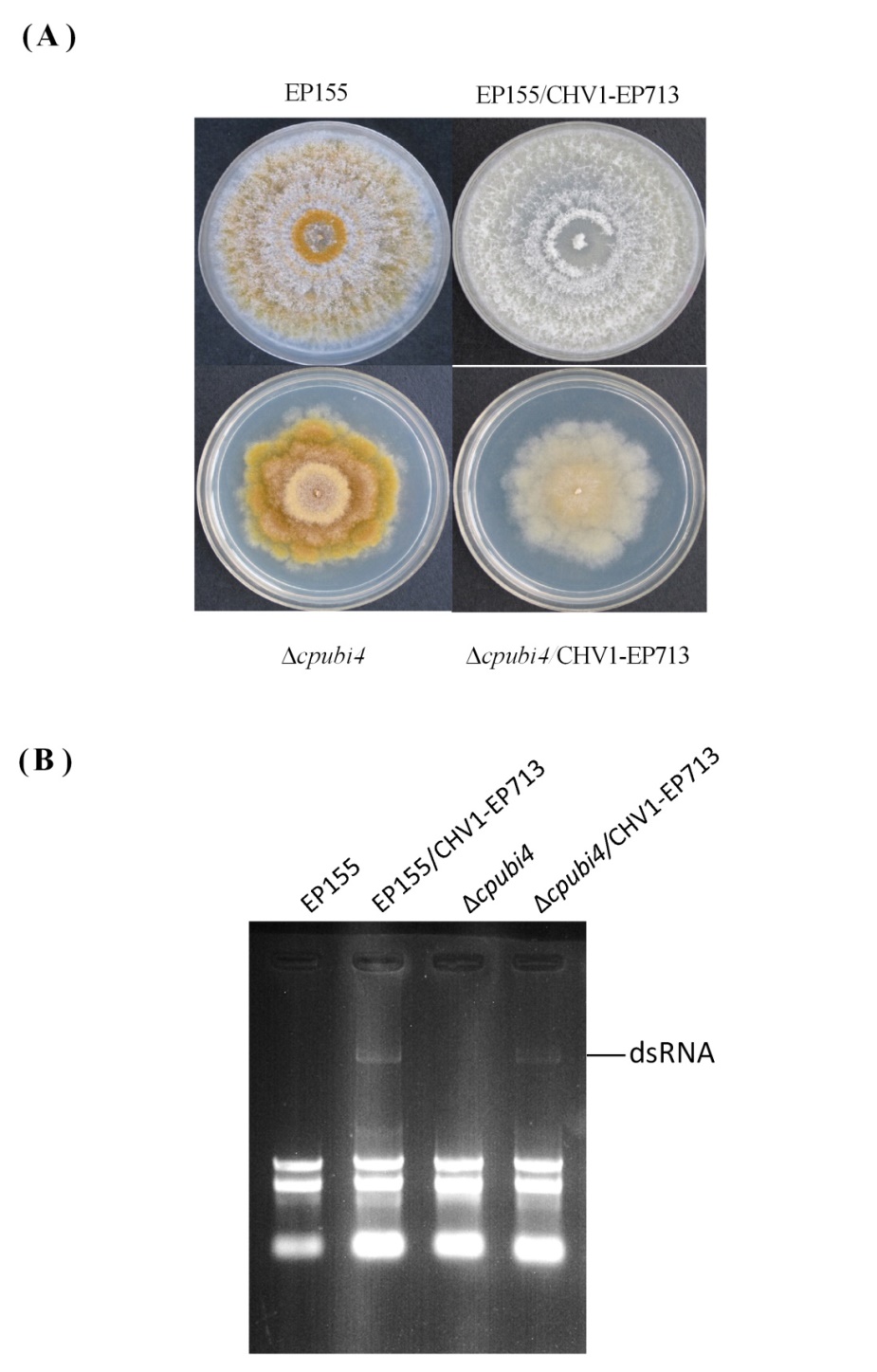


Supplementary Figure 1. Deletion of *cpubi4* does not inﬂuence the accumulation of the hypovirus RNA A, Colony morphologies of wild-type strain EP155, hypovirus infected strain EP713, *cpubi4* disruptants Δ*cpubi4* and the hypovirus infected *cpubi4* disruptant strain Δ*cpubi4*-CHV1/EP713 on PDA plates. B, Agarose gel analysis of viral double-stranded RNA (dsRNA) isolated from EP155, EP713, Δ*cpubi4* and Δ*cpubi4*-CHV1/EP713.


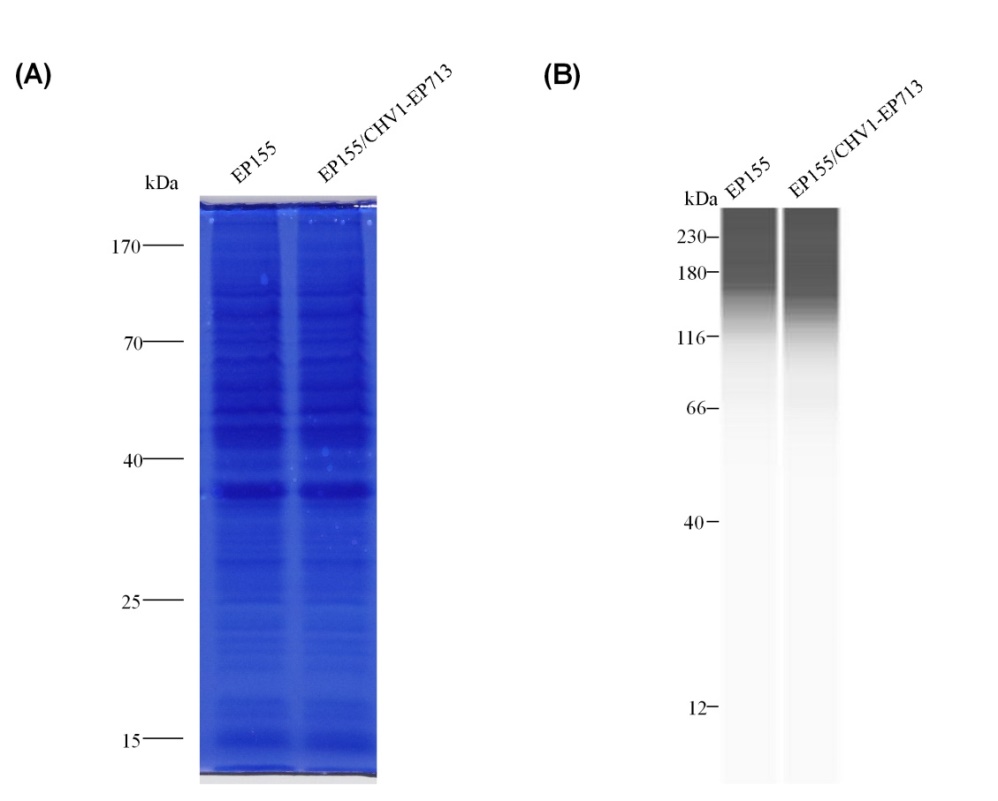


Supplementary Figure 2. Evaluation of the ubiquitylomes of wild-type EP155 and hypovirus infected strain EP155/CHV1-EP713. A, SDS-PAGE. Loading amount was 40 µg of total protein extracted from 3-days old culture in EP. The gel was stained with Coomassie solution. B, Immunal blot analysis on a Simple Western system with ubiquitin-specific antibody. An amount of 4 µg of total protein was loaded for each lane. Signals were stronger with the high molecular weight proteins, likely due to multi ubiquitination sites in the protein.
